# Complications in Interventional Radiology: the role of clinical governance and iterative hospital systems in quality improvement

**DOI:** 10.1186/s42155-023-00388-5

**Published:** 2023-08-05

**Authors:** Warren Clements, Jim Koukounaras

**Affiliations:** 1 Department of Radiology, Alfred Health, 55 Commercial Rd, Melbourne, 3004 Australia; 2 Department of Surgery, Monash University Central Clinical School, Melbourne, Australia; 3 National Trauma Research Institute, Melbourne, Australia

**Keywords:** Adverse event, Complication, Interventional radiology

## Abstract

As modern Interventional Radiology (IR) evolves, and expands in scope and complexity, it will push the boundaries of existing literature. However, with all intervention comes risk and it is the shared judgement of the risk–benefit analysis which underpins the ethical and legal principles of care in IR.

Complications in medicine are common, said to occur in 9.2% of in-hospital healthcare interactions. Healthcare complications also come at considerable cost. It is estimated that in the UK, prolonging hospital stays to manage complications can cost ₤2 billion per year.

However, complications can’t be viewed in isolation. Clinical governance is the umbrella within which complications are viewed. It can be defined as a broadly integrated and systematic approach to clinical care and accountability, that seeks to focus on quality of healthcare. This concept incorporates complications but acknowledges their interplay within a complex healthcare system in which negative adverse events are influenced by a range of intrinsic and extrinsic factors. It also includes the processes that result from monitoring and learning from complications, with feedback leading to systems-based improvements in care moving forward. The reality is that complications are uncommonly the result of medical negligence, but rather they are an unfortunate by-product of a healthcare industry with inherent risk.

It is also important to remember that complications are not just a number on an audit sheet, but a potentially life-changing event for every patient that is affected. The events that follow immediately from an adverse outcome such as open disclosure are vital, and have implications for how that patient experiences healthcare and trusts healthcare professionals for the rest of their life. We must ensure that the patient and their family maintain trust in healthcare professionals into the future.

Credentialling and accreditation are imperative for Interventional Radiologists to meet existing standards as well deal with challenging situations. These should integrate and align within the structure of an organization that has a safety and learning culture. It is the many layers of organisational clinical governance that arguably play the most important role in IR-related complications, rather than apportioning blame to an individual IR.

## Introduction

As modern Interventional Radiology (IR) evolves, and its minimally-invasive therapies expand in scope and complexity, it will push the boundaries of existing literature [1]. However, with all intervention comes risk which arguably increases as our specialty matures [2], and it is the shared judgement of the risk–benefit analysis which underpins the ethical and legal principles of consent and informed consent [3]. Thus, both patients and IRs always enter into a procedure knowing that there is a possibility of some material risk at all times. In fact, the only way to completely avoid this risk is to never perform an intervention.

Complications in medicine are common. A 2008 systematic review identified 8 different studies and a range of complications from 74,485 medical records. The authors showed in-hospital adverse events in 9.2% of patients [4]. However, this figure may be even higher in some subpopulations and countries. The REASON study from Australia assessed complications after surgery in older patients and showed that up to 25% of patients may experience some form of complication within the first 30-days after surgery [5]. Healthcare complications also come at considerable cost. It is estimated that in the UK, prolonging hospital stays to manage complications can cost ₤2 billion per year [6]. In the IR space, studies on complications are minimal often limited to case reports with a particular novel teaching point. In addition, there are a relative lack of healthcare registries amongst IR societies and health networks to monitor our outcomes more broadly, compared to established specialties like surgery. This means that governance processes like reporting of complications are left to the individual practitioner.

In 2017, Filippiadis et al. published the Cardiovascular and Interventional Society of Europe (CIRSE) complications classification system [7], and similar systems exist in other societies. These systems were designed to reduce the interpretability of negative outcomes and allow uniform documentation for governance, legal, and research purposes. The CIRSE complication system uses an ordinal numerical grading system of 1–6, placing weight on complications which needed additional treatment, additional time in hospital, permanent sequelae, or result in death [7].

However, complications can’t be viewed in isolation. Clinical governance is the umbrella within which complications are viewed. It can be defined as a broadly integrated and systematic approach to clinical care and accountability, that seeks to focus on quality of healthcare [8]. This concept incorporates complications but acknowledges their interplay within a complex healthcare system in which negative adverse events are influenced by a range of intrinsic and extrinsic factors [8]. It also includes the processes that result from monitoring and learning from complications with feedback leading to systems-based improvements in care moving forward. The reality is that complications are uncommonly the result of medical negligence, but rather they are an unfortunate by-product of a healthcare industry with inherent risk [9].

It is also important to remember that complications are not just a number on an audit sheet, but a potentially life-changing event for every patient that is affected. The events that follow immediately from an adverse outcome are vital, and have implications for how that patient experiences healthcare and trusts healthcare professionals for the rest of their life. We must ensure that the patient and their family maintain trust in healthcare professionals into the future.

This review will consider the complications more broadly with a focus at a clinical governance level, including strategies required for IR systems to provide high-quality care, and data-driven processes that ensure the delivery of care remains of the highest possible quality as healthcare evolves, acknowledging that complications will always occur even in high functioning teams.

### Learning from experience: acute systems implications

A complication can be defined as a secondary health event that aggravates an existing condition [10]. However, in medicine we also know that sometimes healthcare interactions can be negative but outside this scope. The term “adverse event” is a different way to approach complications, and includes patient perception of an undesired event, even those which may not cause harm or events which may not be directly related to the initial treatment [11]. In this context, pain after a uterine fibroid embolisation procedure may be what an IR would consider within the range of normal, but if the patient expectations were at odds with this outcome then this may be considered an adverse event, and impact on their overall patient journey.

In IR, adverse events may take on many forms [12–14], including the following:

- Technical / device failure: For example, failure of a closure device.
- Haemorrhage: For example, groin haematoma post-arterial intervention or iatrogenic arterial perforation during small-vessel navigation.
- Infection: For example, endometritis post uterine fibroid embolisation, in spite of prophylactic antibiotics and aseptic technique.
- Radiation-induced: For example, skin redness after prolonged and difficult hepatic transarterial chemoembolisation.
- Pain: For example, unexpected severe pain after a procedure such as radiologically-inserted gastrostomy.
- Leak: For example, unintended intraperitoneal leak of bile after percutaneous transhepatic cholangiogram.
- Time delay: For example, elective procedure delayed by many hours due to a concurrent emergency, leading to patient waiting whilst remaining fasted.
- Medication error: For example, wrong dose of unfractionated heparin given during an angiogram.
- Other: There are the potential for many other adverse events in IR given the broad range of procedures and multiple points along the patient journey that can be affected.

Adverse events in IR are traditionally associated with individual errors, and this is perpetuated by legal systems which seek to find individual accountability for a plaintiff [15]. It is important to recognise that adverse events including direct complications are not usually the result of medical negligence, and that these factors are notoriously difficult to measure given varied interpretability of medical records [16, 17]. However, psychologist James Reason analysed errors in a range of high-risk situations and concluded that failures are almost never caused by an individual – rather, accumulation of multiple smaller errors and system flaws [18]. As an example in IR, an adverse event such as dissection during a complex embolisation procedure might be viewed as an individual fault. But in reality, it could be due to any single (or combination) of smaller issues such as time pressure, hunger, tiredness from a previous overnight call-in, poor-quality equipment, workplace hazards such as lack of space for radial access, poor fluoro quality from an old angiography machine, and/or a lack of team to support and assist during a procedure. Other factors such as variant or pathologic anatomy may also play a role. It is quick and easy for a system to blame the individual IR, and even natural for the IR to blame themselves, but this is not usually the correct process in complex environments such as healthcare.

Immediately after a complication or adverse event has been managed and the patient stabilised, there are specific processes that should be performed. Arguably the most important is open disclosure. This can be defined as a process which follows from unintended harm during healthcare, which includes an apology and a discussion on investigation and improvement for the future [19]. This process is underpinned by the morals and ethics in which we practice our craft and is expected by our patients [20]. While this has traditionally been left to individuals to perform at their discretion, increasingly hospitals are requiring this interaction to be a formalised process with a level of documentation. It has even formed part of legislation in our own state of Victoria, Australia including an amendment to the Health Services Act 1988 (Vic) [21]. As humans, it may be natural to be concerned that open disclosure is an admission of guilt. However, if you frame open disclosure as an acknowledgement of the fact and of regret that the patient has suffered harm, rather than as an apology from an individual, it can be easier to accept. This is a fine balance particularly in countries where medicolegal implications of practice are more common [20]. While media and legal implications from complications have been reported, it is not clear to what degree the open disclosure process contributed to this, and it should not be a deterrent from our ethical obligations of beneficence and transparency [22]. Medicolegal societies discuss the difference between open disclosure and admission of liability which are defined differently in legislation, the nuances of which extend beyond the scope of this particular article [23]. Following from open disclosure, it is important to debrief with those involved to identify and record processes which have failed, and may benefit from review or improvement. This will be discussed in the following section.

### Organisational components of dealing with complications

Organisations have an obligation to provide a high-quality workplace. Having defined quality processes has been shown to improve rates of complications, and how individuals and organisations respond to complications [24]. Major stakeholders in this process include government, healthcare professionals such as IRs, and patients – after all, these stakeholders all carry a different form of vested interest in improving quality of care.

One way to look at the framework of quality is the Donabedian model [25]. While this model was proposed in 1966, the core concepts still underpin how modern healthcare is approached. This and similar iterative frameworks suggest that outcomes are related to the interplay between the healthcare system, patients, and several processes [25]. Using this framework within the IR context, complications or adverse events in IR (outcomes) can be said to be related to the underlying healthcare structure (physical equipment and governance characteristics like protocols) as well as processes (training, accreditation, and credentialling).

In assessing the physical structure, there needs to be a minimum standard of acceptable equipment available so that organisations can equip IRs to meet a standard of care. Many colleges and societies outline frameworks on which this can be judged. Locally, the Royal Australian and New Zealand College of Radiologists (RANZCR) recently published its first Standards of Practice for Interventional Radiology which outlines what it defines as appropriate governance, infrastructure, equipment, workforce, clinical care, safety, and incident reporting [26]. Guidelines such as these can be used as a base, which hospitals must meet to benchmark their own structure. Thinking according to the framework of Donabedian, failure to meet structural standards of practice guidelines means that patient outcomes will be directly affected. As an example, if imaging infrastructure was below a published standard at an institution and fluoroscopy quality poor, this may have a direct impact on an ability to navigate a microcatheter to the appropriate site and risks an adverse event such as dissection or non-target embolisation.

Similarly, a framework of protocols for care are equally important within a structure. This may mean that treatments are performed in line with pre-written pathways that are evidence-based and agreed on by multiple different stakeholders. Such protocols encourage appropriate and timely patient referrals, imaging and care according to a set standard. As an example, at the authors’ institution, a protocol exists for management of splenic injury after trauma [27]. This protocol allows junior staff to know when to refer to IR and as such our outcomes of splenic salvage are above many published reference standards [27]. This guideline helps to prevent a potential situation where patients may not be referred even though they have a high-grade injury, and may subsequently need a splenectomy for re-bleed that could have been potentially avoided.

Organisational processes are equally important in accounting for adverse events and complications in IR. It is widely acknowledged that IR training will vary across hospital networks, hospitals have special interest areas which reflect their catchment, and hospitals may have a workflow that reflects healthcare structure in their state or country. Thus, it is important for a set of training standards to ensure that a baseline level of training and competency is achieved in all graduating specialist practitioners regardless of their training site. Extending from this is credentialling, which is a process of analysing training and competency against a standard. The European Board of Interventional Radiology (EBIR) is one example of a widely used credentialling process in IR [28]. The use of tools by a hospital to analyse training, competency, and accreditation means that processes are in place to set a standard for their patients. If an IR is placed in a position to perform a duty for which they are not trained, credentialled, or accredited, and an adverse event occurs, then it is clear that the organisational structure is directly involved in the negative outcome. Consider sites where IRs (as opposed to interventional neuroradiologists or INRs) are asked to perform emergency clot retrieval in the setting of stroke. While this may be within the skillset of many IRs, the specific training and level of competency required is not currently held by the majority in our specialty. Therefore training, accreditation and credentialling are key to preventing adverse outcomes.

The use of hospital-based quality improvement tools is now an expected standard of care, but also falls within modern quality indicators and standards of practice for individual IRs. The most widely used example of this are risk management reporting programs, where clinical incidents such as complications and adverse events can be logged within a system. This provides a way of broad data collection as well as sentinel reporting which in itself is a valuable aid. Such systems allow for incidents to be independently reviewed. These reviews rarely have the intention of apportioning blame, but rather are a transparent process to identify if any inherent organisational processes failed or were suboptimal, and whether these could be improved to prevent a similar adverse outcome in the future. These processes also allow patients to have the assurance that complications are not ignored or downplayed, but are investigated appropriately.

Another process is to analyse and incorporate feedback within a local department quality improvement program. This may allow IRs to modify their practice or local guidelines based on past experiences. While complications will always occur, the ability of a service to show how it responds to past events and to prevent them in the future, is a true indicator of quality. These valuable processes can even be mimicked at a society level with the use of anonymised and confidential complications sessions within scientific meetings. Such sessions, which for example are held at the CIRSE annual congress, allow all IRs to learn from experiences and reflect on what could be done differently at their institution to prevent this from happening. A valuable tool here is the development of guidelines, including ensuring that they expire and are periodically renewed. Such guidelines allow for updating against rapidly-evolving evidence and ensure that steps reviewed in a risk-based pathway can be re-integrated in a prevention mindset. The use of “time-out” processes, which are based on the early concept of surgical checklists, is an example where these concepts were implemented after adverse events in order to prevent potential future incidents of consent or wrong-site operating [29].

Implementation science can be defined as the integration of new thoughts, technology, and processes based on evidence-based data and research [30]. This is an important concept, and is appropriate to consider when discussing organisational components of responding to complications in IR. When using reporting tools or auditing, data is generated which allows sites to benchmark against themselves, their peers, and other hospitals or health networks. By looking at complications broadly they can be compared to existing evidence-based practice guidelines and this provides a hospital the opportunity to review and improve its practice.

### Organisational culture

Organisational culture is a set of values and deep assumptions that underpins the way a healthcare system operates [31]. It is often over-simplified and under-appreciated. However, in reality practitioners including IRs approach their duties within a culture, and this culture will always frame their practice. This is because IRs cannot work in isolation, and culture will affect the remainder of their team impacting peri-operative management and follow-up processes. Within culture, is an important subset of “safety culture”, which includes how an organisation responds to and learns from complications [32]. Things that make a strong safety structure within an organisation include:

- Leadership commitment to safety.
- Organisational responsibility for safety.
- Acknowledging the role of peer-support in safety and complications.
- Systems in place to monitor and respond to complications, and to learn from errors.

It is a vast contrast when considering how an organisation may view an adverse event or complication occurring in IR, depending on its culture [33]. In a blame culture, the IR can be made to feel like it was their fault alone and left to deal with it themselves. This can negatively affect them both physically and mentally, and may then affect future patients they treat as they “second-guess” their training and skillset. In contrast, an organisation with a progressive safety culture may acknowledge their role in governance errors, provide support, work as a team, and approach the patient openly and collaboratively. The individual IR is not personally shamed, but rather investigations are aimed at putting in place processes to prevent a repeat incident and the IR is supported in returning to their normal duties.

### Learning from complications as an individual

While organisational structures form a key to why complications occur and how they are managed, as individual IRs it is also important that we consider internal factors that contribute to complications. Some of these factors include:

- Knowledge of the intervention: the individual IR perception of treatment indication, its risk–benefit profile, and the magnitude of material risks. This includes the current challenges and controversies, as well as standing within current literature.
- Self-efficacy: their own sense of their skill and training regarding the intervention being performed.
- Individual commitment to their organisation: how the IR aligns with the general organisational culture, and the degree of reciprocal commitment. This includes their feeling towards the organisational stance on technology, innovation, and access to equipment.
- Personal safety culture: overall attitudes to complications, including their belief of the open disclosure process, and their ability to provide patient-centric communication.
- Access to resources: this includes IR accessing a wider team, inpatient beds, and support of junior staff. Resources also include time such as non-clinical time and annual leave.
- Access to knowledge: whether the IR is supported in continuing professional development activities, including knowing the latest techniques, broad thinking on how to respond to complications, and opportunities to learn at conferences.

The above intrinsic factors that IRs need to consider are still also linked with their organisation. However, IRs can also link with wider support services which includes professional societies and colleges. By utilising resources from these groups, support can be gathered from a wider community but still remain within a niche. An example may be a complication of arterial rupture occurring during pulmonary angioplasty, which is a procedure not commonly performed [34]. By leveraging on the wealth of resources available from IR societies, support can be gathered from other IRs in the world and/or existing published guidelines on the topic. Such data can be used to inform the open disclosure process and help decide whether this was a sentinel event or within accepted risk profile. Hospitals, societies, and colleges can work collaboratively with the individual IR to implement any prevention processes that arise during an investigation.

### The patient experience

Whilst considering the many intrinsic and extrinsic factors that are at play during complications in IR procedures, we must always remember that patients are the reason we are here. We are ethically bound by principles that include beneficence and non-maleficence.

In considering the previously discussed concepts of adverse events and feedback, it is important to remember that the patient perception of response to errors may be as important as the actual response. One of the principles of the open disclosure dialogue is acknowledging how the patient feels, and showing the patient that adverse events are taken seriously. This has implications for the remainder of the healthcare journey that patient will experience within their life. By providing an organisational structure that makes them feel respected after a complication, and by the IR using individual qualities such as compassion and empathy, we can show that patient that they are welcome back even though their journey may not have been as initially intended during this visit. This can be linked into a formal patient feedback process, which works even better when linked to a dedicated patient advocacy team and involves the patients’ family.

One final aspect of patient-focussed care that can be linked to complications is the concept of public reporting. Currently, hospitals report a range of healthcare indicators to governments and some of this is publicly available [35]. Whilst individual events are not appropriate to be released, the use of broad and de-identified data on outcomes may help to provide transparency of a health network’s performance. This potential public scrutiny may encourage the health system to evolve and better deal with complications. Concurrently, it will empower patients to use this data to take their care to a system that they feel is likely to support them. Whether this is feasible within IR remains to be seen as it is not yet studied. But it is important that we think of these concepts as we deal with complications, because legislation evolves rapidly and public healthcare transparency is an important topic.

## Conclusion

Complications and adverse events in medicine are common and are a significant cost to healthcare. There are several clinical governance factors that contribute to complications in IR including why they occur and how we deal with them. IRs should consider important aspects of training and accreditation, and should integrate within an organisation which has a safety and learning culture they align with. As discussed in this review, it is the many layers of organisational clinical governance that arguably play the most important role in IR-related complications, rather than apportioning blame to the individual IR.

## Data Availability

N/A – no data generated for this review article.

## Abbreviations

IR: Interventional Radiology
INR: Interventional Neuroradiology
CIRSE: Cardiovascular and Interventional Society of Europe
EBIR: European Board of Interventional Radiology
RANZCR: Royal Australian and New Zealand College of Radiologists
